# Carpets

**Published:** 1871-08

**Authors:** 


### CARPETS

Are not as cleanly and healthful as a painted, waxed, or well-washed floor, but as they make a room look so much more cosy, give it a more finished appearance, and keep it warmer in winter, giving at the same time greater quiet about a house, they will continue to be used by all who can afford them. Very many of the best houses in England and on the Continent have only strips of carpets alongside the beds in their chambers, and only the centre of their parlors covered.

Persian and Turkey carpets are the best in the world, but from their expensiveness, seldom find their way to this country.

Next to these in quality is the Aubusson, made in France, entirely of wool, woven in one piece for the size of the room.

Axminsters are third best, the warp being entirely of linen, the woolen thread being so woven in as to conceal the linen entirely.

Wilton carpets look like velvet, because the loops are cut entirely through.

Brussels come next; more of these are sold than of other kinds, composed of linen and worsted, one thread of linen being above, another below the woolen.

Tapestry velvet has the loops of worsted cut like the Wilton, and differs from tapestry, only in this.

Tapestry Brussels is woven in a common loom and printed in the warp.

Ingrain, Kidderminster, or Scotch carpets are called three-ply when they have three thicknesses, and two-ply when of two, and are made by the intersection of two or more cloths of different colors; the thread being so arranged that the figures on both sides are alike, only reversing the colors; these are thirty-six inches wide; the velvets, Brussels, etc., are twenty-seven inches.

The United States carpet is perhaps the cheapest and most durable of all, having been known before now to last through two generations; it is the old-fashioned "rag-carpet," which needs no description, except by the rising generation in cities. Our grandmothers made them by saving every woolen rag in the family, cutting it in strips less than half an inch broad, sewing the ends together, and filling it in on the common loom on cotton or linen. Among the happiest associations of many a reader who has passed half a century, are those connected with the memory of the rag-carpet.

